# Natural history of Barth syndrome: a national cohort study of 22 patients

**DOI:** 10.1186/1750-1172-8-70

**Published:** 2013-05-08

**Authors:** Charlotte Rigaud, Anne-Sophie Lebre, Renaud Touraine, Blandine Beaupain, Chris Ottolenghi, Allel Chabli, Helene Ansquer, Hulya Ozsahin, Sylvie Di Filippo, Pascale De Lonlay, Betina Borm, Francois Rivier, Marie-Catherine Vaillant, Michèle Mathieu-Dramard, Alice Goldenberg, Géraldine Viot, Philippe Charron, Marlene Rio, Damien Bonnet, Jean Donadieu

**Affiliations:** 1 AP-HP, Registre français des neutropénies chroniques sévères, Centre de référence des déficits Immunitaires Héréditaires, Service d’Hémato-oncologie Pédiatrique Hôpital Trousseau, Paris, France; 2 Assistance Publique-Hôpitaux de Paris (Hôpital Necker-Enfants Malades, Service de Génétique) and INSERM U781, Université Paris Descartes, Paris, France; 3 Service de Génétique, Centre Hospitalo-Universitaire, Saint Etienne, France; 4 AP-HP, Laboratoire de Biochimie, Hôpital Necker-Enfants Malades et Université Paris Descartes, Paris, France; 5 Service de Néonatalogie, Centre Hospitalo-Universitaire, Brest, France; 6 Service d’Onco-Hématologie pédiatrique, Hôpital des enfants Hôpitaux Universitaires, Genève, Switzerland; 7 Service de Cardiologie pédiatrique, Hôpital Louis Pradel, Centre Hospitalo-Universitaire, Lyon, France; 8 AP-HP, Centre de référence des maladies métaboliques, Hôpital Necker-Enfants Malades, Université Paris Descartes, Paris, France; 9 Service de Pédiatrie, Centre Hospitalier, Béziers, France; 10 Service de neuropédiatrie, Hôpital Gui de Chauliac, Centre Hospitalo-Universitaire, Montpellier, France; 11 Service de Médecine Pédiatrique, Centre Hospitalo-Universitaire, Tours, France; 12 Service de Génétique, Centre Hospitalo-Universitaire, Amiens, France; 13 Service de Génétique, Centre Hospitalo-Universitaire, Rouen, France; 14 AP-HP, Service de Génétique, Hôpital Cochin, Paris, France; 15 Centre de référence pour les maladies cardiaques héréditaires,CHU Pitié-Salpêtrière, Paris, France; 16 National reference Center for Complex Congenital Heart Defects-M3C, AP-HP, Université Paris Descartes, Sorbonne Paris Cité, Paris, France

**Keywords:** Barth syndrome, Cardiomyopathy, Neutropenia, *TAZ* gene, Cohort

## Abstract

**Background:**

This study describes the natural history of Barth syndrome (BTHS).

**Methods:**

The medical records of all patients with BTHS living in France were identified in multiple sources and reviewed.

**Results:**

We identified 16 BTHS pedigrees that included 22 patients. *TAZ* mutations were observed in 15 pedigrees. The estimated incidence of BTHS was 1.5 cases per million births (95%CI: 0.2–2.3). The median age at presentation was 3.1 weeks (range, 0–1.4 years), and the median age at last follow-up was 4.75 years (range, 3–15 years). Eleven patients died at a median age of 5.1 months; 9 deaths were related to cardiomyopathy and 2 to sepsis. The 5-year survival rate was 51%, and no deaths were observed in patients ≥3 years. Fourteen patients presented with cardiomyopathy, and cardiomyopathy was documented in 20 during follow-up. Left ventricular systolic function was very poor during the first year of life and tended to normalize over time. Nineteen patients had neutropenia. Metabolic investigations revealed inconstant moderate 3-methylglutaconic aciduria and plasma arginine levels that were reduced or in the low-normal range. Survival correlated with two prognostic factors: severe neutropenia at diagnosis (<0.5 × 10^9^/L) and birth year. Specifically, the survival rate was 70% for patients born after 2000 and 20% for those born before 2000.

**Conclusions:**

This survey found that BTHS outcome was affected by cardiac events and by a risk of infection that was related to neutropenia. Modern management of heart failure and prevention of infection in infancy may improve the survival of patients with BTHS without the need for heart transplantation.

## Background

Barth syndrome (BTHS; MIM 302060) is a recessive X-linked mitochondrial disorder first described by Barth *et al.* in 1983 in a large pedigree of Dutch patients [1]. This syndrome is characterized by cardiomyopathy, neutropenia, skeletal myopathy and growth delay. While clinical symptoms are usually present early in infancy [2], the age at presentation and the features of BTHS vary significantly among patients [3]. BTHS is caused by mutations in the *TAZ* gene, located in Xq28 [4]. This gene encodes the Tafazzin protein, which is involved in the remodelling of cardiolipin, an essential component of the mitochondrial inner membrane that is necessary for proper function of the respiratory chain [5,6]. No genotype-phenotype correlation has been described to date [7]. Currently, there are 151 recorded cases of BTHS worldwide [8] and several surveys have been published in the last 10 years. The largest one analysed the growth and cardiac outcome of 73 patients, extending the observations in previous studies [9,10] and Additional file 1: Table S1 offers a review of all available surveys [1,2,9-18]. Clinical heterogeneity, the rarity of the syndrome and a lack of data regarding the epidemiology and natural history of BTHS prompted us to conduct a national study in France to analyse clinical and biological features of the disease as well as disease outcomes.

## Design and methods

### Patients

This was an observational longitudinal retrospective study. The study included all French patients diagnosed with BTHS between January 1, 1983 and August 1, 2012. A diagnosis of BTHS was suspected when cardiomyopathy and neutropenia were present and BTHS was confirmed by identification of a mutation in the *TAZ* gene or when i) the patient was a male with clinical signs of BTHS and had an informative cardiolipin profile [19] or ii) the patient was a male maternal relative of a confirmed BTHS patient with a *TAZ* mutation who presented with clinical signs of BTHS. To ensure that our enrolment was as complete as possible i.e. that we included all BTHS cases in France, patients were recruited from several sources. First we identified all cases that were already registered in the French severe chronic neutropenia registry. Then we contacted all paediatricians who belonged to the French Society of Paediatric Haematology and Immunology, all French paediatric cardiologists in the French Society of Congenital and Paediatric Cardiology and all genetics laboratories that perform the analysis of the *TAZ* gene*.* We asked these sources to identify their patients with known BTHS or patients who presented with dilated cardiomyopathy and neutropenia. Patients with an informative cardiolipin profile were recruited from the Biochemistry Laboratory at the Necker Enfants Malades Hospital. The French Patients and Parents Association was solicited to complete the enrolment. Finally, the national database of death certificates (http://www.cepidc.vesinet.inserm.fr) was checked in order to identify patients whose immediate or underlying cause of death was Barth syndrome. Once a case was identified, the patient was included in the registry, and data were collected from the patient’s medical charts. All patients and/or their parents gave written informed consent for inclusion in the registry.

### Clinical investigations

The following data were systematically extracted from the patients’ files: demographic and physical characteristics, cardiac evaluations, haematological parameters, gross motor delays, medications, genotype and need for nutritional support. Laboratory evaluations such as cardiolipin profiles and urinary organic acid and plasma amino acid profiles were also recorded. Prematurity was defined as gestational age less than 37 weeks. Patients were considered to have severe intrauterine growth retardation (IUGR) if the birth weight was below the 3rd percentile for the gestational age. Age at presentation was defined by the age at which the first pathological manifestations were noted that led to the diagnosis of BTHS.

### Cardiac evaluation and definitions

We collected all available echocardiogram records for each patient. The echocardiogram performed at the time of diagnosis was considered to be the first echocardiogram at the age of diagnosis. The left ventricular end diastolic diameter (LVEDD) and the left ventricular mass (LV mass) were analysed according to age and body surface area, and z-scores were calculated according to standard distribution [20]. The left ventricular ejection fraction (LVEF) was also recorded. Dilated cardiomyopathy (DCM) and hypertrophic cardiomyopathy (HCM) were defined by LVEDD and by LV mass z-score greater than +2 standard deviations (SD), respectively. Information about hospitalisation for heart failure, therapy for heart failure (including the use of mechanical ventilation), inotropic drugs, heart transplantation and cause of death were recorded. Electrocardiograms (ECG) and 24-hour ECG/Holter monitor records were also collected. The corrected QT interval (QTc) was calculated using Bazett’s formula.

### Haematological definitions and neutropenia-related complications

The first complete blood count (CBC) performed for the patient was considered the initial CBC. Baseline CBCs were considered if they were collected during routine consultations, with the exception of periods in which granulocyte-stimulating factor (GCSF) therapy was given. Neutropenia was defined by an absolute neutrophil count (ANC) below 1.5 × 10^9^/L, and severe neutropenia was defined by an ANC below 0.5 × 10^9^/L. The results of bone marrow smears were reviewed to look for myeloid arrest, which was defined as in previous studies [21].

Severe infections were defined as those that would be life-threatening without appropriate antibiotic or antifungal therapy and that required medical supervision or hospitalisation. These events were exhaustively recorded in the patients’ medical records. Minor infections were stomatological infections, ear, nose and throat (ENT) infections, or bronchitis.

### Metabolic investigations

Cardiolipin analysis was performed using standard liquid chromatography and mass spectrometry according to a published method [19]. Urine organic acids and plasma amino acids were investigated by GC-MS and HPLC-ninhydrin colorimetry, respectively, according to standard methods. As a reference population for plasma arginine and ornithine levels, we used 12,837 samples from 10,618 patients without known inherited metabolic diseases or parenteral nutrition that were treated during the previous 5 years at Necker Hospital.

### *TAZ* gene testing

The patients or their parents gave written informed consent for genetic testing. Genomic DNA was extracted from blood using standard procedures. The coding sequence and exon-intron boundaries of the *TAZ* gene were amplified by polymerase chain reaction (PCR) using primers and conditions that are described elsewhere [4,22]. The *TAZ* mutations were numbered as recommended by the Human Genome Variation Society (http://www.hgvs.org/) using the reference sequence NM_000116.3.

### Statistical analysis

Stata software version 10 was used for all statistical analyses. Range (minimum-maximum) values and median values were used to represent the distribution of quantitative variables. The number of births per year in France (metropolitan areas excluding Reunion Island, French Polynesia and French Antilles) was extracted from records at the Institut National de la Statistique et des Etudes Economiques (http://www.insee.fr). The incidence at birth was assumed to satisfy a Poisson distribution. For survival analysis, the endpoint was death. The period taken into account was the time interval from birth until death (if the patient died) or, alternatively, from birth until the last examination (if the patient was still alive at the end of the study period). The Kaplan-Meier method was used to estimate survival rates. Survival was compared between groups using the log-rank test, and the Cox model was used for multivariate analysis. The cut-off date was September 1, 2012. Foetal data were not included in the statistical analysis for survival.

## Results

### Demographic data

We analysed data from 22 subjects in 16 pedigrees (Additional file 2: Figure S2). The patient characteristics are shown in Table 1. Six individuals that were referred as potential BTHS patients in different pedigrees were excluded because of a lack of clinical data (families 4 and 11; Additional file 2: Figure S2). Medical information about the 22 patients was collected from a total of 26 sources, which illustrates the complexity of the medical management of this disease. All patients were Caucasians, including three patients of Tunisian origin (family 11) while the others were of European ancestry. One patient was a girl (UPN 5938) for whom cytogenetic analysis showed mosaicism for X monosomy and for a ring X chromosome; her characteristics were reported elsewhere [22]. The BTHS diagnosis was confirmed by the presence of a *TAZ* mutation in 18 patients (73%), and the 4 other patients had a presumptive diagnosis based on an informative cardiolipin profile in 1 and clinical signs of BTHS in a proven pedigree in 3. At least 1 affected relative was found for 14 patients (64%). The median age at presentation was 3.1 weeks (range, 0–1.4 years), and the median age at the last follow-up for the 11 living patients was 4.75 years (range, 3–15 years). Cardiomyopathy was the presenting symptom in 16 (73%) patients, with 3 cases diagnosed prenatally. Infection was the presenting symptom in 4 patients (18%). The other modes of onset are summarised in Table 1. There was only one preterm newborn (born at 36 weeks of gestation). The median birth weight was 2770 g (range, 2180–3730 g) and 7 patients had severe IUGR (32%). The estimated incidence at birth, calculated for 1995–2008, was 1.5 cases per million births, with 95% confidence interval limits of 0.6 and 2.3 cases per million births.

**Table 1:** Clinical characteristics of the 22 patients in the French Barth syndrome cohort

** UPN**	**Family number**	**Sex**	**Age at diagnosis (y)/mode of onset**	**Age at last-follow-up (y)/vital status (cause of death)**	**Positive family history**	**Genotype**	**Mother with proven *TAZ *mutation**	**Median baseline ANC (x10**^**9**^**/L)**	**Clinical signs** **of HF**	**SF/EF at diagnosis (%)**	**LVEDD z-score at diagnosis**	**LV mass z-score at diagnosis**	**LVNC**	**SGA new** **born**	**Age of walking (months)**	**3-MGCA**	**Informative CL profile**
5938	1	F	0.09/Cardiomyopathy	2.56 D (Septic choc)	No	Del exon 1-5	Yes	0.85	Yes	9/24	3.1	4.6	Yes	Yes	24	No	Yes
5930	2	M	0.01/Cardiomyopathy	7.82 L + Heart Transplanted at 0.65 y	No	Exon 2/c.143delinsGG/p.Glu48fsX	Yes	1.93	Yes	20/38	4.5	−0.4	Yes	No	24	No	Yes
5940	3	M	0.07/Infection	3.45 L	No	Exon 3/c.280C > A/p.Arg94Ser	Yes	0.98	Yes	N/A	0.7	1.4	No	No	20	Yes	Yes
7101	4	M	1.37/Cardiomyopathy	3.20 L	No	Exon 3/c.281G > A/p.Arg94His	Not mutated	2.90	Yes	19/30	13	N/A	No	No	N/A	Yes	Yes
5932	5	M	0.08/Cardiomyopathy	13.5 L	Yes	Exon 4/c.356T > G/p.Val119Gly	Yes	1.79	Yes	25/58	2.8	N/A	Yes	No	N/A	Yes	Yes
7112	6	M	0.69/Infection	12.61 L	No	Exon 6/c.478A > T/p. Lys160X	N/A	0.30	No	34/70	−0.7	−0.9	No	No	18	Yes	Yes
5941	7	M	0.05/Cardiomyopathy	0.22 D (Acute HF)	Yes	Del exon 6-11	N/A	0.77	Yes	14/N/A	N/A	N/A	No	Yes	N/A	Yes	Yes
5804	8	M	0.11/Cardiomyopathy	11.02 L	Yes	Del exon 6-11	Not mutated	0.61	Yes	31/61	6.6	N/A	No	Yes	18	Yes	N/A
5937	9	M	0.13/Infection	0.28 D (Acute HF)	No	Exon 8/c.589G > A/p.Gly197Arg	Yes	1.00	Yes	10/N/A	6.6	N/A	No	Non	N/A	N/A	Yes
5931	10	M	IU/Cardiomyopathy	0.47 D (Acute HF)	No	Exon 8/c.589G > T/p.Gly197Trp	Yes	0.52	Yes	12.3/23.8	7.5	6.4	No	Yes	N/A	Yes	Yes
5939	11	M	IU/Cardiomyopathy	1.81 D (Electromechanical dissociation 12 hours after heart transplant)	Yes	Exon 8/c.646G > A/p.Gly216Arg	Yes	13.63	Yes	25/N/A	1.3	N/A	No	Yes	N/A	No	Yes
7105	11	M	IU/Cardiomyopathy	0.41 D (Acute HF)	Yes	Not tested	Yes	0.66	Yes	20/N/A	1.3	N/A	No	No	N/A	N/A	N/A
7104	11	M	Birth/Cardiomyopathy	0.16 D (HF)	Yes	Exon 8/c.646G > A/p.Gly216Arg	Yes	N/A	Yes	N/A	N/A	N/A	No	No	N/A	N/A	N/A
5933	12	M	0.10/Sepsis	0.10 D (Septic choc)	Yes	Not tested	Yes	0	No	N/A	N/A	N/A	No	No	N/A	N/A	N/A
5934	12	M	0.71/Cardiomyopathy	0.76 D (Acute HF + fever)	Yes	Del exon 8-9	Yes	2.50	Yes	13.7/25.6	12.7	7.8	No	No	N/A	N/A	Yes
5935	13	M	0.17/Cardiomyopathy	0.47 D (Acute HF + fever)	Yes	Exon 9/c.659_660dupGTCC/p.Leu221fsX	Not mutated	0.70	Yes	16/35	7.4	2	No	No	N/A	No	Yes
5936	13	M	Birth/Cardiomyopathy	2.17 L	Yes	Exon 9/c.659_660dupGTCC/p.Leu221fsX	Not mutated	1.47	Yes	30/N/A	3.3	8.6	Yes	No	N/A	No	Yes
6042	14	M	1.7/Growth delay	4.33 L	Yes	Intron 9/c.700-1G > A/p. ?	Yes	0.72	Yes	8.5/16.3	8.4	3.5	Yes	Yes	N/A	No	N/A
7100	15	M	Birth/Hypoglycaemia	4.75 L	Yes	not detected	not detected	0.97	Yes	12.8/28.3	10.2	4.3	Yes	Yes	21	Yes	Yes
7102	15	M	Birth/Cardiomyopathy	0.43 D (HF)	Yes	Not tested	N/A	2.88	Yes	N/A	N/A	N/A	No	No	N/A	N/A	N/A
7111	16	M	Birth/Cardiomyopathy	3.33 L	Yes	Intron 10/c. 778-1G > T	N/A	3.82	Yes	16/36.1	1.9	3	Yes	No	18	Yes	Yes
7110	16	M	Birth/Cardiomyopathy	8.56 L	Yes	Intron 10/c. 778-1G > T	N/A	4.28	Yes	N/A	N/A	N/A	Yes	No	12	Yes	Yes

*UPN*, unique patient number; *M*, male; *F*, female: *y*, years; *IU*, in utero; *L*, living; *D*, dead; *HF*, heart failure; *TAZ*: TAZ gene; *N/A*, not available; *SF*, shortening fraction; *EF*, ejection fraction; *LVEDD*, left ventricular end of diastole diameter, *LV mass*, left ventricular mass; *LVNC*, leftventricular noncompaction; *SGA*, small for gestational age; *3-MGCA*, 3-methylglutaconic aciduria; *CL*, cardiolipin.

### Genetics

Fourteen different mutations in the *TAZ* gene were found in 15 different pedigrees. No mutation was identified in 1 pedigree. These mutations included 1 nonsense mutation, 2 frameshift mutations, 6 amino acid substitutions, 3 large deletions (more than one exon deleted) and 2 splicing defect mutations. Some of these mutations have been reported previously [7,14,22-27]. Excluding the large *TAZ* deletions, the mutations in our patients were in exon 2 (n = 1), exon 3 (n = 2), exon 4 (n = 1), exon 6 (n = 1), exon 8 (n = 4), exon 9 (n = 1), intron 9 (n = 1) and intron 10 (n = 1). The mutations are summarized in Table 2. The 13 mothers of the 17 patients were tested for *TAZ* mutations: 9 were carriers with somatic *TAZ* mutations, and 1 had proven somatic mosaicism (unpublished data). All of the mothers appeared to be healthy, but no biological tests or heart ultrasounds were performed.

**Table 2:** **The *TAZ *mutations of the 22 patients in the French Barth syndrome cohort**

**Location**	**Nucleotide sequence change**	**Protein effect**	**Type of mutation**	**Number of pedigrees**	**References**
Exons 1-5	del exon 1-5		Exon deletion	1	[22,23,28]
Exon 2	c.143delinsGG	p.Glu48fsX	Frame shift mutation	1	This report
Exon 3	c.280C > A	p.Arg94Ser	Missense mutation	1	[24]
Exon 3	c.281G > A	p.Arg94His	Missense mutation	1	[26]
Exon 4	c.356T > G	p.Val119Gly	Missense mutation	1	This report
Exon 6	c.478A > T	p.Lys160X	Nonsense mutation	1	[28]
Exons 6-11	del exon 6-11		Exon deletions	2	[28]
Exon 8	c.589G > A	p.Gly197Arg	Missense mutation	1	[7,14,27]
Exon 8	c.589G > T	p.Gly197Trp	Missense mutation	1	[28]
Exon 8	c.646G > A	p.Gly216Arg	Missense mutation	1	[28]
Exons 8-9	del exon8-9		Exon deletion	1	This report
Exon 9	c.659_660dupGTCC	p.Leu221fsX	Frameshift mutation	1	This report
Intron 9	c.700-1G > A	p. ?	Splicing defect	1	[28]
Intron 10	c. 778-1G > T	p.?	Splicing defect	1	This report
			Undetermined mutation*	1	
				16	

### Haematological features and infectious events

**Figure 1:**
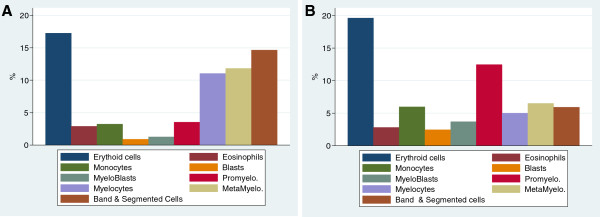
**Bone marrow smear differential cell count data, including the percentage of the indicated granulocyte precursors. A**) Normal bone marrow. **B**) Bone marrow from patients with Barth syndrome (mean of 4 bone marrow counts). A granulopoietic blockage was observed in the Barth syndrome bone marrow samples, but there were no additional morphologic abnormalities.

The median initial absolute neutrophil count (ANC) was 1.3 × 10^9^/L (range, 0–6.4 × 10^9^/L) and 4 patients (18%) had an initial ANC ≤ 0.5 × 10^9^/L. The median age at first CBC was 1.2 months (range, 0–2.2 years). During follow-up, a median of 8 CBC values per patient was available (range, 1–46 values). The median baseline white blood cell count (WBC) was 7.6 × 10^9^/L (range, 3.4–15.5 × 10^9^/L) and the median baseline ANC was 0.98 × 10^9^/L (range, 0–13.6 × 10^9^/L). In all cases with serial CBCs, the ANC fluctuated over time without any detectable regular variation. Sixteen patients (73%) had an ANC <0.5 × 10^9^/L at least once, and 2 (9%) had a median ANC <0.5 × 10^9^/L. The median absolute lymphocyte count (ALC) was 4.3 × 10^9^/L (range, 1.9–10.5 × 10^9^/L) and the median absolute monocyte count (AMC) was 1.1 × 10^9^/L (range, 0.5–4.3 × 10^9^/L). The median haemoglobin (Hb) level was 11.2 g/dL (range, 9.7–15.1 g/dL) and the median platelet count was 336 × 10^9^/L (range, 194–59110^9^/L). Transient anaemia (≤7 g/dL) was observed in 2 patients and was probably caused by iterative blood sampling during intensive care hospitalisation. At baseline, 5 bone marrow smears were available, 2 (40%) of which showed typical promyelocyte-myelocyte maturation arrest. Even in samples in which there was not a complete myeloid arrest, we observed a greatly decreased proportion of myelocytes, metamyelocytes and neutrophils, and an increased proportion of promyelocytes (Figure 1). GCSF was used to prevent infection in 6 patients. Only 2 patients received long-term GCSF therapy. In the other 4 cases, GCSF was used “on demand” i.e. when an infection occurred. Four patients received antibiotic prophylaxis.

We recorded 94 infectious events, including 10 severe and 84 mild infections. The 10 severe infections were observed in 5 patients (23%). These infections were cellulitis in 3 cases, pneumonia in 3 cases and septicaemia in 4 cases. Septicaemia led to septic shock in 3 cases and was associated with another infection in 2 cases. The median age of the first severe infection was 3.7 months (range: 0.2–30.7 months).

Bacterial pathogens were identified in 6 cases: *Pseudomonas aeruginosa* in two cases and *Staphylococcus epidermidis, Klebsiella oxytoca, Enterobacter cloacae and Mycoplasma pneumonia* in 1 case each. We did not find any infections caused by a fungal agent. Although 84 mild infections were recorded, some, such as bronchitis or upper respiratory tract infections that lacked proof of viral infection, can have serious consequences. In all, 16 patients (73%) were hospitalised during infectious episodes, and there were a total of 56 hospitalisations, 11 of which required ICU admission. Four patients died during an infectious episode, 2 of septic shock and 2 of cardiac failure associated with high fever without bacterial documentation. Acute stomatitis occurred in just 1 patient. No chronic periodontal disease was reported. One patient had repeated episodes of diarrhoea.

### Cardiology features

Twenty patients (91%) had symptomatic cardiomyopathy. One of the oldest patients of the cohort (UPN 7112, age 12.6 years) never showed any clinical signs of cardiomyopathy. The other patient died early (at 1 month) of septic shock and suspicion of acute myocarditis, but autopsy results did not confirm cardiomyopathy (UPN 5933).

The 20 patients (91%) with clinical evidence of cardiomyopathy were each hospitalised at least once for heart failure episodes, and there were a total of 54 hospitalisations for heart failure. In 11/54 (20%) of the episodes, heart failure worsening was due to infection. During follow-up, 16 of the 20 patients (73%) needed inotropic support, 14 (64%) needed invasive ventilation and 11 (50%) were treated with both inotropic agents and invasive ventilation at least once. One patient needed a paediatric ventricular assist device before cardiac transplant. Four patients were on a cardiac transplant list, and 2 of them underwent cardiac transplant. UPN 5930 is alive 7 years after transplant, and UPN 5939 died the day after transplantation of cardiac arrest due to electromechanical dissociation. Two of the patients on the transplant list never received a heart transplant: UPN 7105 died of heart failure, and UPN 7101 recovered and was withdrawn from the transplant list. Finally, 9 patients died of heart failure.

Four of these patients never received any treatment for heart failure: 2 never needed any treatment (UPN 7112, aged 12.4 years at last follow-up, and UPN 5932, aged 13.4 years at last follow-up); 1 died early of septic shock and had no previous signs of cardiomyopathy; and 1 had no documented medication in his medical records but did have evidence of chronic heart failure. Of the 20 patients who presented with documented heart failure, most of them (n = 16) received aggressive treatment for heart failure, including inotropic agents (n = 16), mechanical ventilation (n = 14) or both (n = 11).

Among the 9 untransplanted living patients, 7 needed inotropic support and/or mechanical ventilation at least once. These 7 patients received a total of three to five other medications for chronic heart failure (Table 3) when the heart failure was at its worst. At last follow-up, 4 patients did not need any cardiac medication. Medical management of cardiac failure of BTHS patients has improved since 2000, with more widespread use of beta-blockers for chronic heart failure and milrinone for acute heart failure.

**Table 3:** Therapy received by the 22 patients in the French Barth syndrome cohort

**Medications**	**n (%)**
ACE-I	16 (72.7%)
β-Blockers	9 (40.9%)
Digoxin	11 (50%)
Diuretics	17 (77.3%)
Anticoagulants	5 (22.7%)
Aspirin	5 (22.7%)
Antibiotic prophylaxis	4 (18.2%)
GCSF	6 (27.2%)
Other therapies	12 (54.5%)
	22 (100%)

*ACE-I*, angiotensin-converting enzyme inhibitors; *GCSF*, granulocyte colony stimulating factor.

An echocardiogram at diagnosis was available for 17 patients. At diagnosis, the median LVEDD z-score was 4.5 (range, -0.7–12.7), the median LV-mass z-score was 3.5 (range, -0.4–8.6), the median SF was 16% (range, 8.5–34%) and the median EF was 32.5% (range, 16.3–70%). Six of the 11 patients who had information available at diagnosis had associated dilated cardiomyopathy (DCM) and hypertrophy cardiomyopathy (HCM) (54.5%), 2 had DCM only and 1 had HCM only. In addition, 7 patients (32%) had prominent trabeculations of the LV, either on echocardiogram or on MRI, and were considered to have left ventricular noncompaction (LVNC).

**Figure 2:**
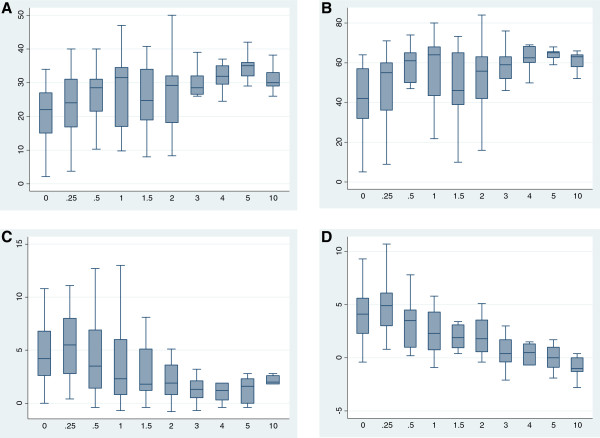
**Box plots showing the distribution of cardiology parameters as determined by ultrasound according to age.** Age is shown for the following categories: 0, birth to age 3 months; 0.25, between 3 months and 6 months; 0.5, between 6 months and 1 year; 1, between 1 and 1.5 years; 1.5, between 1.5 and 2 years; 2, between 2 and 3 years; 3, between 3 and 4 years; 4, between 4 and 5 years; 5, between 5 and 10 years; 10, >10 years old. The heart indicators are as follows: **A**) shortening fraction (SF), reported as %; **B**) ejection fraction (EF), reported as %; **C**) z-score of the left ventricular end diastolic diameter (LVEDD); **D**) z-score of the left ventricular mass (LV mass).

During follow-up, a total of 266 echocardiograms were performed, with a median of 12.5 echocardiograms per patient (range, 1–31 echocardiograms). All of the 15 patients with LV mass z-score and LVEDD z-score that could be measured on the same echocardiograms during follow-up had both DCM and HCM. The evolution of the echocardiographic parameters with age showed that LVEDD and LV mass z-scores varied according to the same patterns (Figure 2). Specifically, LVEDD (Figure 2C) and LV mass (Figure 2D) were elevated and increased during the first 6 months of life. After 6 months, LVEDD and LV mass tended to decrease until the age of 2 years and remained stable after that. In our series, we were not able to follow the evolution of these cardiac parameters later in life because of the limited number of older children in the series. Similarly, the LVEF was altered in the first 6 months of life (Figure 2B) and tended to improve thereafter. There were some cases in which LV systolic function recovered fully. Notably, there was important inter-individual variation, especially in the narrow age categories, as shown by wide distribution ranges and interquartile intervals for all of the cardiology parameters.

Twenty patients had records of at least one ECG, and a total of 46 ECGs were recorded. All of them showed normal sinus rhythms. Repolarisation abnormalities were found in 36 ECG s from 17 patients (77.3%). Repolarization abnormalities were predominantly ST flattening and T-wave inversion. The median QTc was 440 ms (range, 360–530 ms), and only 25% of the cases presented values within the normal range if QTc <420 ms was considered normal while 25% presented with QTc > 460 ms. All patients had normal QRS duration. Eight patients completed at least one 24-hour Holter monitor. There was no evidence of atrial or ventricular arrhythmia.

### Skeletal myopathy and other clinical features

The age for walking was available for 8 patients who survived until the age of 2.5 years, and the median age for walking was 19 months (range, 12 –24). All of the patients that were able to walk ambulated independently; none of them needed a wheelchair. Muscle biopsy samples were available for 7 patients and showed lipid storage myopathy in 6 of them. None of the patients had severe mental retardation, and all patients showed normal academic progression. Seven patients needed long-term enteral nutritional support. Five of the 10 living patients were below the 3rd percentile in weight for their age, and 5 were below −3 SDs in height for their age.

### Biochemical data

**Figure 3:**
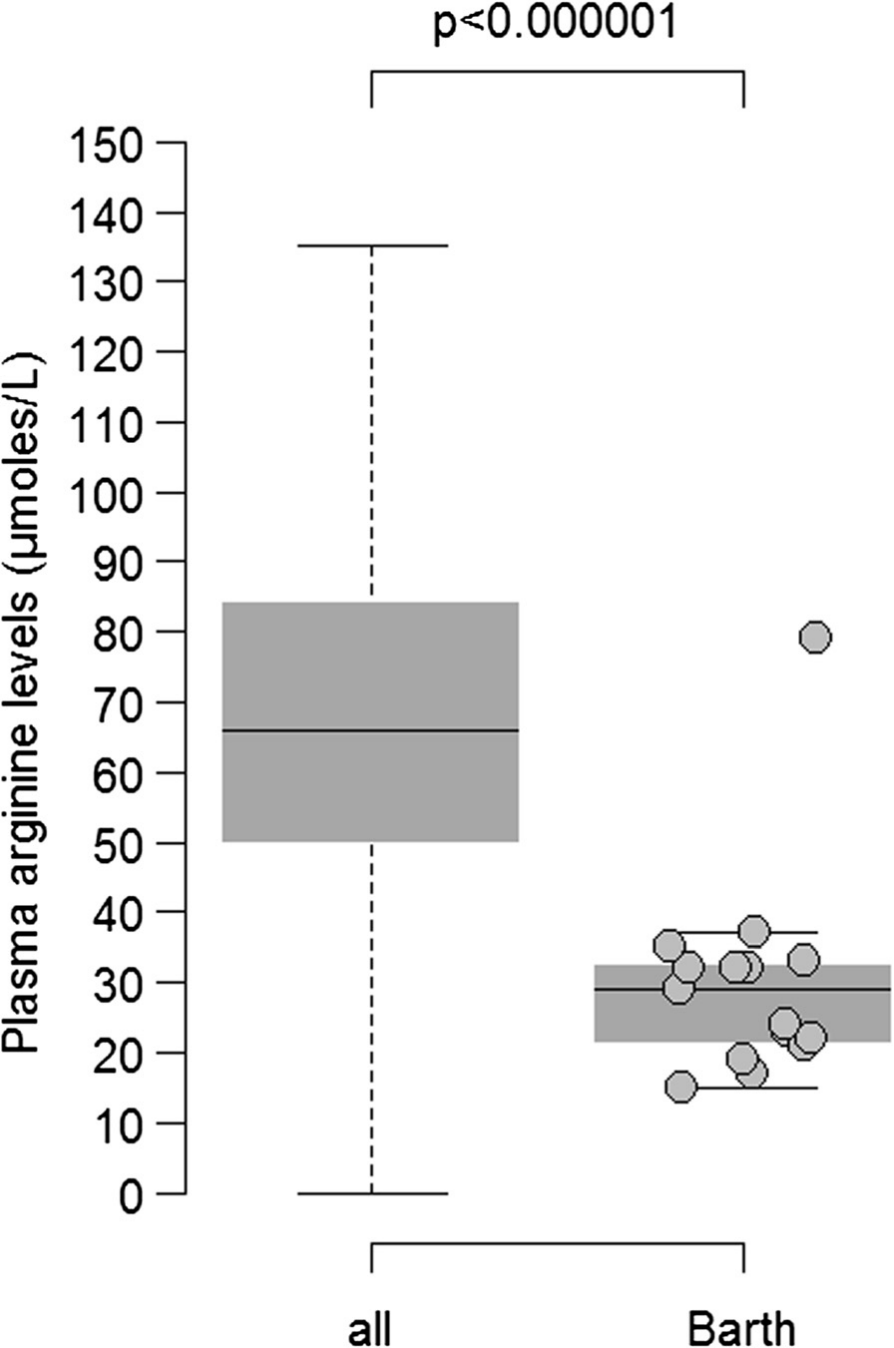
**Plasma arginine levels.** Levels are shown in samples from patients in the French Barth syndrome cohort (15 samples) and in samples from an entire hospital patient population (12,828 samples). The plot shows that 95% of the samples from Barth Sd patients fall below the lower 25-percentile of randomly selected patients. Y-axis: concentration in micromoles/litre. From left to right, arginine levels in the hospital population (“all”) and in Barth syndrome patients (“Barth”). The box plots show the median values (horizontal bars), the 50th percentile around the median (box), and the full range of the 95th percentile distribution (dotted vertical lines). Circles indicate the values from individual samples from Barth syndrome patients. p-values: Wilcoxon rank sum test.

Three-methylglutaconic aciduria was found in 8 of the 16 patients tested (50%) at diagnosis. A cardiolipin study was performed in 16 patients, in lymphoblasts for 1 patient, in platelets for 1, and in cultured fibroblasts for the others. All of the screened patients had an informative profile that showed low cardiolipin (CL) levels, the presence of monolysocardiolipin (MLCL) and an elevated MLCL/CL ratio. In all cases in which both a CL study and *TAZ* testing were available, the two results were in agreement. A total of 15 plasma amino acid profiles were available for 8 patients. These 8 patients showed low arginine levels as compared to a cohort of over ten thousand patients that had no known metabolic diseases (p < 10^-6^, Wilcoxon rank sum test; Figure 3). Thus, a reduced arginine level appears to be a feature that is found consistently in a sizeable fraction of BTHS patients. These results are consistent with unpublished work by Dr. Kelley (Kennedy Krieger Institute).

### Survival

**Figure 4:**
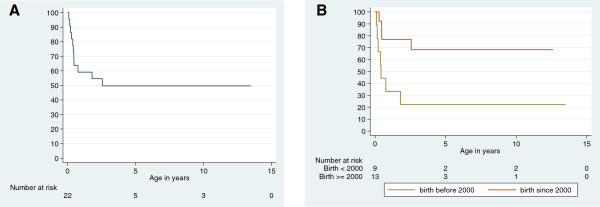
**Overall survival and survival according to birth year of the French Barth syndrome cohort. A**) Kaplan-Meier plot and 95% confidence intervals showing the overall survival of the French Barth syndrome cohort. Time is expressed in years since birth. **B**) Kaplan-Meier plot showing the survival of the French Barth syndrome cohort according to birth year (before and in or after 2000). Even though the total number of patients is quite limited, the difference in survival is both important (survival at 5 years: 22% for patients born before 2000 and 70% for patients born in or after 2000) and statistically significant (p = 0.009). This suggests that recent progress in the management of heart dysfunction may improve the survival of patients with BTHS.

Eleven of the 22 patients died during the study period. The median age at death was 5.1 months (range, 1.2–30.7). Death was caused by cardiac failure in 9 of 11 patients and was associated with a febrile episode, probably of viral origin, in 2 of the 9 patients. Death was due to septic shock in the other 2 patients. Figure 4A shows that mortality was high during the first three years of life, especially in infants. The 5-year survival rate was 51% (95%CI: 29.1%–69.3%), while a plateau was observed later. The following potential prognostic factors for survival were studied in univariate analysis: heart function parameters at diagnosis (including shortening fraction, ejection fraction and LV mass), neutrophil count, platelet count, haemoglobin levels at diagnosis, birth weight and the year of birth (before vs. in or after 2000). We stratified the birth year as before vs. in or after 2000 because several new drugs were introduced for the medical management of heart dysfunction during this period, including milrinone and beta-blockers. While the parameters of the heart ultrasound at diagnosis showed no obvious impact on survival, two prognostic factors stood out: the severity of neutropenia at the first CBC and the birth year. Patients with absolute neutrophil counts <0.5 × 10^9^/L had a 1-year survival rate of 25%, while those in whom ANC was above 0.5 ×10^9^/L had a 1-year survival rate of 68%. The 4 patients with severe initial neutropenia died by age 5 years, while 62% of patients without severe neutropenia survived past their 5th birthdays (p = 0.004). Survival was also significantly different depending on the year of birth: the 5-year survival rate was 22% in patients born before 2000 vs. 70% in patients born in or after 2000 (Figure 4B). When these two prognostic factors were analysed together in a Cox model, they both remained associated with mortality.

## Discussion

Here we described the French national cohort of patients with BTHS. We identified 22 cases in 16 pedigrees and performed a longitudinal data analysis that provides a detailed description of the cardiac and haematological parameters and the outcomes of the disease. This study suggests that BTHS is an extremely rare disease with an estimated incidence of 1.5 cases per million live births. This incidence rate was calculated for the 1995–2008 period and our analysis included all patients that had a confirmed BTHS diagnosis during this period. However, we cannot exclude the possibility that some patients with milder clinical courses who may not have required medical attention or who died suddenly may have remained undiagnosed. Such patients were not included in our survey. The incidence rate is close to a previous preliminary estimate of 1 case per 500,000 live births [28], considerably lower than a recent report from the UK of 1:140,000 [8], although our confidence intervals were larger. Our result is the first estimate based on a comprehensive national survey that combined several sources of information, including both cardiology and haematology paediatric networks, as well as a parents’ association directory. However, our BTHS French cohort did not include any adult cases and included a very limited number of adolescents, whereas patients in these age categories are present in other clinical studies [9,29-31] and are registered with the Barth Syndrome Foundation (BSF). The lack of enrolment of teenagers and adults may thus represent an underestimation of the prevalence of the disease and limit our observations of morbid complications in adults. However, this should not affect the calculation of the incidence at birth, since our enrolment is likely exhaustive for the time period during which the incidence was estimated.

Patients with BTHS are predisposed to premature death. It is quite difficult to analyse the mortality rate in BTHS using data in the literature. The initial description [1,12,24,32] found a high and very early mortality. In a recent study of 6 pedigrees, 6 deaths were observed among 13 proven BTHS cases; 5 patients died at birth or before the age of 1 month, while one died at age 7 years [27] (Additional file 1: Table S1). However, other studies demonstrate the possibility of long-term survival in patients with BTHS, although it is unclear whether this is the result of better medical management or if the improvement in survival is related to better identification of cases thanks to improvements in diagnosis [33]. The severity of the initial BTHS cases that were described in the literature resulted in estimates of almost 90% mortality in young patients with BTHS. This contrasts with several cross-sectional studies of young adults with BTHS [9,10,31] that show relatively few deaths in children or young adults (5 deaths at ages 5.8, 7.4, 16.2, 23.0 and 25.4 years among 73 patients). Our study reconciles this apparent contradiction, showing that mortality in BTHS peaks in the first years of life and then falls. Our study, which has limited information about teens and adults, did not allow us to evaluate the late mortality that was observed in another study [9]. In all cases, the outcome is driven by cardiac events associated with acute infections. Some of these infections were related to neutropenia, but simple viral infections may be complicated by cardiac failure. In our survey, babies who survived the first year showed good survival with limited morbidity. Notably, when we compared survival in babies born before and after 2000, we found that the median age at death did not change but that overall survival was improved (Figure 4B). We interpret this improvement to be a consequence of advances in the management of acute and chronic heart failure in children. In particular, the systematic use of beta-blockers and modern inotropic drugs like milrinone have decreased the incidence of heart failure, both isolated or during an infectious episode, which is the main cause of death in BTHS. Importantly, this improvement in survival did not correlate with heart transplantation. Only 2 patients in our survey underwent heart transplantation, one of which showed long term survival. Indeed, management of cardiomyopathy is a key and vital issue in BTHS. The most frequent type of cardiomyopathy in BTHS is dilated cardiomyopathy [10]. Here, we described an unusual phenotype i.e. dilatation of the left ventricle with hypertrophied walls. This hypertrophied-dilated phenotype is also observed in respiratory chain defects [34] and in LVNC. Even if it is non-pathognomonic because it can also be seen in respiratory chain defects, this cardiac phenotype in a male infant is highly suggestive of BTHS and should prompt the physician to search for other phenotypes associated with the syndrome, particularly low ANC. LVNC was observed in only a third of our patients, which is a lower proportion than reported previously [10]. This might be due to the retrospective nature of our study in which echocardiograms were not reviewed to search for prominent trabeculations. The evolution of cardiac functional parameters i.e. LVEDD z-score, LV mass z-score, SF and EF, correlated with the shape of the survival curve. Cardiac functional parameters also correlated with very poor cardiac outcomes in the first year of life. After two years of age, the cardiac function of the patients continued to improve and was normal thereafter in most cases. The ECG data matched the findings of the BTHS cohort [10], showing an increased median QTc and showing that approximately 25% of patients had prolonged QTc values >460 milliseconds. Conversely, we did not document any atrial or ventricular arrhythmias in our patients; nevertheless, one possible explanation is that arrhythmia in BTHS is generally reported in patients older than 11 years, an age that was marginally represented in our cohort [10,16]. Although this study provides data on the evolution of cardiac parameters in BTHS, we do not have information for teenagers, young adults or adults as we did not recruit any patients that were over 13 years old.

Neutropenia is a classical characteristic of BTHS and represents an important clue for BTHS diagnosis. In about 20% of cases, the presenting symptoms of BTHS are infections facilitated by neutropenia. During follow-up, we observed that 19 patients had episodes of neutropenia, while 3 had no episodes of neutropenia; however, the latter had a limited number of blood examinations (1 or 2) during follow-up.

The prevalence of neutropenia in this study is higher than the previously reported rate of 25% [10], but is close to the prevalence reported in two studies for the BTS registry (69% [9], and 90% [8]) in which neutropenia was self-reported and is consistent with the historical description of BTHS [1,33].

Indeed, we observed that neutropenia was an intermittent feature in our series and could sometimes be diagnosed only during follow-up with routine blood sampling. None of our patients exhibited cyclic neutropenia as described in the 1990s [1,26,33,35]. ANC is unpredictable in BTHS in that a single normal ANC count in male infants with cardiomyopathy does not exclude BTHS. Despite the frequency of neutropenia, the clinical consequences are difficult to assess. Neutropenia in BTHS could be life threatening by allowing severe infections or by facilitating infections that lead to heart failure. Notably, although neutropenia seemed to respond well to GCSF treatment, two episodes of severe infection, including one episode of septic shock, occurred while patients were on GCSF therapy. Despite chronic neutropenia, we did not record any chronic gingivostomatitis or periodontal disease, in contrast to the other manifestations of congenital neutropenia [36]. Even if rare or underdiagnosed in our survey, such oral manifestation is a useful indication for GCSF. Bone marrow examination showed mild or profound maturation arrest, as described for other congenital neutropenias such as those associated with the ELANE mutation [21]. In addition, none of the living patients had secondary leukaemia; this distinguishes BTHS from other subtypes of congenital neutropenia, even though the number of reported cases of BTHS is limited.

Our study provides additional information about the growth characteristics of patients with BTHS. We reported that about 30% of newborns with BTHS had a birth weight below the 3rd percentile. The proportion of severe IUGR in BTHS is not known and remains controversial [28,37]. In a recently survey of 73 cases, 20% of BTHS patients were reported to have a birth weight below 2.5 kg [9]. In fact, when analysing all BTHS case reports that report birth weight (n = 45), about 30% of the patients show a birth weight that is below the 3rd percentile, a proportion that is higher than that of the normal population and which is consistent with our findings. In addition, we observed that the heights of half of the patients over 5 years old fell below the 3rd percentile. We found low arginine levels in most of the plasma samples we tested, which could lead to a reduced growth rate (Figure 3). This finding is consistent with a partial defect in the Krebs cycle that could lead to arginine being metabolized into alpha-ketoglutaric acid at a higher than normal rate. Whatever the mechanism(s) involved, arginine depletion may have detrimental effects due to limitations in protein synthesis [38]. This may support the use of arginine supplementation as an auxiliary therapy for improving the growth rate of BTHS patients.

Signs of skeletal myopathy in patients have often been described by clinicians [39], but no objective data are available. However, we noticed a slight gross motor delay and an increased median age of walking. There was no associated mental retardation.

Disease-causing mutations have been found in all exons of the *TAZ* gene [4,40,41]. The *TAZ* mutations in our cohort have been reported in previous studies [7,14,22-27] and/or in the BSF *TAZ* mutations database [28]. Allelic heterogeneity is high in BTHS, and the intrafamilial variability of expression is high. Thus, no genotype-phenotype correlation has been observed in any series, including ours [7,27]. Half of the mothers that were tested for *TAZ* mutations were carriers. One of the mothers we tested had 2 affected children (UPN 5935 and UPN 5936) but Sanger sequencing did not show any somatic *TAZ* mutations. However, the mother had somatic mosaïcism [unpublished data] and most probably gonadal mosaicism, as suspected in a Japanese family with BTHS [41].

Notably, both in our cohort and in other BTHS patients [42], 3-methylglutaconic aciduria was not found consistently in BTHS patients, even when the assay was repeated in a given patient; thus, this result must not be considered mandatory for a definitive diagnosis of BTHS.

Finally, the natural course of this disease illustrates the benefits of an interdisciplinary approach. In particular, a diagnosis of cardiomyopathy in a newborn or infant should lead to repeated tests to determine whether low ANC is present. The reverse is also true: It may be worth adding cardiac evaluation to the workup of the diagnosis of a chronic neutropenia suspected to be congenital. This diagnostic work-up should be performed in close cooperation with geneticists and metabolic disease specialists.

## Abbreviations

ACE-I: Angiotensin-converting enzyme inhibitor; ALC: Absolute lymphocyte count; AMC: Absolute monocyte count; ANC: Absolute neutrophil count; BSF: Barth Syndrome Foundation; BTHS: Barth syndrome; CBC: Complete blood count; CL: Cardiolipin; DCM: Dilated cardiomyopathy; GCSF: Granulocyte colony-stimulating factor; G6PC3: Glucose-6-phosphatase catalytic subunit 3 gene; Hb: Haemoglobin; HCM: Hypertrophic cardiomyopathy; IUGR: Intrauterine growth retardation; LV: Left ventricle; LVEDD: Left ventricle end diastolic diameter; LVEF: Left ventricle ejection fraction; LVNC: Left ventricular noncompaction; MLCL: Monolysocardiolipin; PCR: Polymerase chain reaction; QTc: Corrected QT interval; SD: Standard deviation; SF: Shortening fraction; TAZ: *TAZ* gene; UPN: Unique patient number; WBC: White blood cell count

## Competing interests

The authors declare no competing interests.

## Authors’ contributions

A steering committee that included JD, DB, ASL, CO, AC and MR planned the study. ASL and RT carried out the molecular genetic studies, and CO and AC carried out the metabolic studies. DB analysed the data pertaining to the cardiac evaluations. CR collected the data and drafted the manuscript. JD was responsible for the statistical analysis and for the organization of the French SCN registry. All authors read and approved the final manuscript.

## Supplementary Material

Additional file 1: Table S1 Review of all published studies about Barth syndrome patients. Click here for file

Additional file 2: Figure S1 Family trees showing the 16 Barth syndrome pedigrees reported in this study. Click here for file
