# Supplementary material for: Natural history of Barth syndrome: a national cohort study of 22 patients
Source: Orphanet J Rare Dis. 2013 May 8;8:70. doi: 10.1186/1750-1172-8-70 (PMC3656783; doi:10.1186/1750-1172-8-70)
Supplement: Additional file 1: Table S1 — Review of all published studies about Barth syndrome patients. [file 1750-1172-8-70-S1.doc]

**Additional file Table S1:** Review of all published studies about Barth syndrome patients

| **Author**  **Publication Year**  **Reference** | **Country** | **Number of patients/**  **number of families** | **Age at presentation** | **Mode of onset** | **Mortality %** | **Cause of death** | **Follow-up** | **Neutropenia % of patients**  **/ANC** | **Bacterial infection, sites** | **GCSF** | **Cardiology event rate** | **SF/EF** | **Type of CM/LVEDD and LV mass** | **LVNC** | **QTc/arrythmia** | **Cardiac treatment** | **Skeletal myopathy** | **Growth retardation** |
| --- | --- | --- | --- | --- | --- | --- | --- | --- | --- | --- | --- | --- | --- | --- | --- | --- | --- | --- |
| Barth  1983  [1] | The Netherlands | 18 patients  1 family | N/A | Known in 3 patients: 1 CM, 1 neutropenia, 1 infection | 94 | Known in 7: 57% HF, 43% infection | Min 0- max 3.5 y | 33%/from 0 to normal number of neutrophils | Pneumonia, sepsis, cellulitis | N/A | 94% | 25% (only 1 SF available) | DCM on autopsy in 6/6/N/A | N/A | N/A | N/A | 53% | Only documented in one patient (height and weight between 3rd and 10th percentile) |
| Kelley  1991  [2] | USA | 7 patients  5 families | Median=4 mo (min birth; max 7 y) | Known in 4 patients: CM | 33 | Both cardiac and infectious death causes. Rates N/A | Median=5 y (min 2_max 32); patient year=61.4 | From 0 to 1 10^9^/L | Noted in 4 patients, unknown sites | N/A | 0% | SF at diagnosis=9.5% (n= 4) | DCM | 1 patient (autopsy) | N/A | digoxin, diuretics, | yes | Yes, height < 3SD |
| Orstavik  1998  [11] | Norway | 7patients  3 families | Median=1.5 mo (min IU; max 9 mo) | 100% CM | 100 | HF 100% | Median=1 mo (min 0_max 16); 1.6 Patient year | 14.20% | N/A | N/A | 7 deaths | N/A | 100% DCM | N/A | N/A | N/A | N/A | N/A |
| Gedeon  1995  [12] | Australia | 6 patients  1 family | Median=2 days (min 0; max 11 days) | CM | 100 | 5 CM and 1 SDIS | Median=0.07 y (min 0_max 0.41) | N/A | N/A | N/A | 6 deaths | N/A | 100% DCM | 16.70% | N/A | Digoxin, diuretics | N/A | N/A |
| Bleyl  1997  [13] | USA | 6 patients  1 family | Median=0.125y (min 0; max 0.58) | CM | 67 | 1 SDIS, 2 HF, 1 cardiac arrest during arrythmia | Median=0.03 y (min 0- max 4.5) | 33% | N/A | N/A | 4 deaths, 1 heart transplant | Median=15.5 (min 7 - max 22); 6 SF measurements available | 100% DCM and 80% HCM | 100% | Arrythmia=75% | Digoxin, diuretics | 16,70% | Yes < 5th p |
| Cantlay  1999  [14] | UK | 5 patients  5 families | Median=4 mo (min 1 mo; max 16 mo) | 20%infection; 80%DCM | 80 | 3 DCM, 1 lymphoma | Median=0.67 y (min 0.16, max 6.5); 12.2 patient year | 80%/median of medians=0.710^9^/L (range from0 to 12) | Sites N/A | N/A | 4 deaths, 1 heart transplant | N/A | 100% DCM | N/A | N/A | N/A | 40% | N/A |
| Rugolotto  2003  [15] | Italy | 3 patients  2 families | Median=3 mo (min 0; max 3 mo) | 33% infection, 67% CM | 0.00 | N/A | Median=4.75 y (min 1.5 - max 14.75); 21 patient year | 100%/ANC from 0.05 to 0.75 10^9^/L) | Nephritis, ENT infection, mastoiditis, stomatitis, groin abcess | 100% | 1 heart transplant (33%) | Median EF=40 (min 20, max 62) | 3 with DCM | N/A | N/A | Digoxine, ACE- I, diuretic | N/A | Yes, from 3rd to 10th percentile in height and weight |
| Spencer  2005  [16] | USA | 5 patients | Median=13 mo (min IU; max 12 y) | CM ? | N/A | N/A | Median=14.9 y (min 1 -max 19 y) | N/A | N/A | 20% |  | Median SF 25 (min 17- max 32) | 3 DCM/median z-LVEDD=4.7 (min 4,3 max 5.5) (7 measurements) | 20% | Ventricular Arrythmia 100% | Digo (100%), IEC (60%), B- (40%), aspirin (10%, Coenz Q (40%) | N/A | N/A |
| Spencer  2006  [10] | USA | 34 patients | Mean=5.5 mo (+/- 7.4) | CM ? | N/A | N/A | Mean age at evaluation=10.2 y range from 1.2 to 22.6 y | 25% with an ANC < 1 10^9^/L | N/A | 25% | N/A | Mean z-score: SF=-2.7/EF=-1.9 | mean LVEDD z=1,9, CMD | 53% with increased trabeculations. | 20% > 460ms/20.6% VA | Digo (64%), IEC (61%), B- (32%), Diuretic (29%), CoQ (22%). | yes, no dependence, z score grip strength=-2.9 (RH) et -2.1 (LH) | Mean weight=15th p, mean height=8th p (15 patients with weight < 5th percentile and 15 with height< 5th percentile) |
| Mangat  2007  [17] | UK | 4 patients | Infancy | CM | 0 | N/A | Current age from 2 to 16 y | 50% | Gastroenteritis, stomatitis, cellulitis, cryptosporidiose, Pneumonia, Peritonitis (aspergillus) | N/A | 4 heart transplant | 3 SF available before heart transplant: median=9 (min 6 - max 14) | DCM/1 z-LVEDD available before heart transplant=7.7) | N/A | N/A | N/A | 75% | Yes, from <2^nd^ to 9th percentile |
| Hastings  2008  [18] | UK | 12 patients  10 families | Infancy | 75%CM, 16.7% motor delay, 8.3% difficulties feeding | N/A | N/A | N/A | 75% | Arthritis, pneumocytosis (post heart transplant),septic shock | 42% | 42% heart transplant | N/A | DCM | N/A | N/A | N/A | 100% | Yes from 0.4 to 10 percentile |
| Roberts  2012  [9] | International | 73 patients | Mean=0.76 ± 1.6 y | 70% CM | 7 pts | 60% HF, 20% sepsis, 20% both HF + sepsis | Range from 0.3 to 28.7 y; mean follow-up: 2.14 ±1.81 y; 156.6 patients years | 69.1% | Stomatitis, pneumonia, other sites | 49.2% | 9 heart transplant | Respectively 71 of SF and 67% of EF z-scores ≥ +3SD; | N/A | N/A | 12% of intra cardiac defibrillator | N/A | 65.7% of delay sitting-up and 71.6%% of delay walking | Height and weight on the 3^rd^ p at 36 mo and near the 50^th^ p after 16 y |

CM: cardiomyopathy; DCM: dilated cardiomyopathy; HCM: hypertrophic cardiomyopathy; HF: heart failure; y: years; mo: months, p: percentile; IU: in utero; LV: left ventricle; LVEDD: left ventricular end diastolic diameter; SF: shortening fraction; EF: ejection fraction ANC: absolute neutrophil count; SDIS: sudden death in infancy syndrome; RH: right hand; LH: left hand; N/A: not available
