# Supplementary material for: Natural history of Barth syndrome: a national cohort study of 22 patients
Source: Orphanet J Rare Dis. 2013 May 8;8:70. doi: 10.1186/1750-1172-8-70 (PMC3656783; doi:10.1186/1750-1172-8-70)
Supplement: Additional file 2: Figure S1 — Family trees showing the 16 Barth syndrome pedigrees reported in this study. [file 1750-1172-8-70-S2.doc]

**Supplementary Figure 1 :** Family Trees of the 16 BTHS Pedigrees

UPN: Unique Patient Number; DCM: Dilated Cardiomyopathy; ?: *TAZ* not tested in potential carrier for BTHS if female or potential BTHS patient if male with suspect death

**Family 1**

Del exon 1-5

**Family 2**

Exon 2

c.143delinsGG

E48fs133X

UPN 5930

Heart transplant ( 0,65 y)

**Family 3**

Exon 3

c.280C>A

p.Arg94Ser

UPN 5940

**?**

UPN 7101

**Family 4**

Exon 3

c.281G>A

p.Arg94His

?

Miscarrage 1st trimester

UPN 5938

?

?

**Supplementary Figure 1 :** Family Trees of the 16 BTHS Pedigrees

UPN: Unique Patient Number; DCM: Dilated Cardiomyopathy ; ?: *TAZ* not tested in potential carrier for BTHS if female or potential BTHS patient if male with suspect death

**Family 5**

Exon 4

c.356T>G

p.Val119Gly

?

?

Death at 2 months

2

?

?

?

?

2 healthy boys

Feticide in the 3rd trimester for DCM

Neonatal death

Death at 2 months

Death at 6 months

UPN 5932

**Family 6**

Exon 6

c.478A>T

p.Lys160X

**Family 7**

Del exon 6-11

v

?

?

?

UPN 7112

?

?

Death at 2 months

UPN 5941

**Supplementary Figure 1 :** Family Trees of the 16 BTHS Pedigrees

UPN: Unique Patient Number; DCM: Dilated Cardiomyopathy; ?: *TAZ* not tested in potential carrier for BTHS if female or potential BTHS patient if male with suspect death

UPN 5804

**Family 8**

Del exon 6-11

UPN 5937

**Family 9**

Exon 8

c.589G>A

p.Gly197Arg

Death at 1 month (Tetralogy of Fallot)

UPN 5931

Feticide in the 3rd trimester

?

**Family 10**

Exon 8

c.589G>T

p.Gly197Trp

?

**Supplementary Figure 1 :** Family Trees of the 16 BTHS Pedigrees

UPN: Unique Patient Number; DCM: Dilated Cardiomyopathy; ?: *TAZ* not tested in potential carrier for BTHS if female or potential BTHS patient if male with suspect death

**Family 11**

Exon 8

c.646G>A

p.Gly216Arg

?

7

7

7 women not tested

?

?

Death at 2 month (DCM)

UPN 7104

UPN 5939

UPN 7105

**Family 13**

Exon 9

c.659_660dupGTCC

p.Leu221fsX

*TAZ* gonadal mosaïcism

**Family 12**

Del exon 8-9

Miscarrage 1st trimester

UPN 5936

UPN 5933

UPN 5934

**Supplementary Figure 1 :** Family Trees of the 16 BTHS Pedigrees

UPN: Unique Patient Number; DCM: Dilated Cardiomyopathy

?

?

?

UPN 6042

Swiss patient

Neonatal diagnosis (Sepsis + DCM)

**Family 14**

Exon 10

c.700-1G>A

p. ?

Isolated developmental retardation

Miscarrage 1st trimester

Abortion

UPN 7100

UPN 7102

**Family 15**

*TAZ* mutation not detected

**?**

?

?

?

**Supplementary Figure 1 :** Family Trees of the 16 BTHS Pedigrees

UPN: Unique Patient Number; DCM: Dilated Cardiomyopathy; ?: *TAZ* not tested in potential carrier for BTHS if female or potential BTHS patient if male with suspect death

**Family 16**

Intron 10

c.778-1G>T

p. ?

UPN 7110

UPN 7111

= Male with suspected BTHS

= Male with BTHS

= Female carrier for BTHS

?
